# HypoxamiRs Profiling Identify miR-765 as a Regulator of the Early Stages of Vasculogenic Mimicry in SKOV3 Ovarian Cancer Cells

**DOI:** 10.3389/fonc.2019.00381

**Published:** 2019-05-14

**Authors:** Yarely M. Salinas-Vera, Dolores Gallardo-Rincón, Raúl García-Vázquez, Olga N. Hernández-de la Cruz, Laurence A. Marchat, Juan Antonio González-Barrios, Erika Ruíz-García, Carlos Vázquez-Calzada, Estefanía Contreras-Sanzón, Martha Resendiz-Hernández, Horacio Astudillo-de la Vega, José L. Cruz-Colin, Alma D. Campos-Parra, César López-Camarillo

**Affiliations:** ^1^Posgrado en Ciencias Genómicas, Universidad Autónoma de la Ciudad de Mexico, Mexico City, Mexico; ^2^Laboratorio de Medicina Translacional y Departamento de Tumores Gastro-Intestinales, Instituto Nacional de Cancerología, Mexico City, Mexico; ^3^Programa en Biomedicina Molecular y Red de Biotecnología, Instituto Politécnico Nacional, Mexico City, Mexico; ^4^Laboratorio de Medicina Genómica, Hospital Regional 1 de Octubre ISSSTE, Mexico City, Mexico; ^5^Departamento de Infectómica y Patogénesis Molecular, CINVESTAV-IPN, Mexico City, Mexico; ^6^Laboratorio de Investigación Translacional en Cáncer y Terapia Celular, Hospital de Oncología, Centro Médico Nacional Siglo XXI, Mexico City, Mexico; ^7^Subdirección de Investigación Básica, Instituto Nacional de Medicina Genómica, Mexico City, Mexico; ^8^Laboratorio de Genómica, Instituto Nacional de Cancerología, Mexico City, Mexico

**Keywords:** ovarian cancer, vasculogenic mimicry, hypoxia, miR-765, VEGFA

## Abstract

Vasculogenic mimicry (VM) is a novel cancer hallmark in which malignant cells develop matrix-associated 3D tubular networks with a lumen under hypoxia to supply nutrients needed for tumor growth. Recent studies showed that microRNAs (miRNAs) may have a role in VM regulation. In this study, we examined the relevance of hypoxia-regulated miRNAs (hypoxamiRs) in the early stages of VM formation. Data showed that after 48 h hypoxia and 12 h incubation on matrigel SKOV3 ovarian cancer cells undergo the formation of matrix-associated intercellular connections referred hereafter as 3D channels-like structures, which arose previous to the apparition of canonical tubular structures representative of VM. Comprehensive profiling of 754 mature miRNAs at the onset of hypoxia-induced 3D channels-like structures showed that 11 hypoxamiRs were modulated (FC>1.5; *p* < 0.05) in SKOV3 cells (9 downregulated and 2 upregulated). Bioinformatic analysis of the set of regulated miRNAs showed that they might impact cellular pathways related with tumorigenesis. Moreover, overall survival analysis in a cohort of ovarian cancer patients (*n* = 485) indicated that low miR-765, miR-193b, miR-148a and high miR-138 levels were associated with worst patients outcome. In particular, miR-765 was severely downregulated after hypoxia (FC < 32.02; *p* < 0.05), and predicted to target a number of protein-encoding genes involved in angiogenesis and VM. Functional assays showed that ectopic restoration of miR-765 in SKOV3 cells resulted in a significant inhibition of hypoxia-induced 3D channels-like formation that was associated with a reduced number of branch points and patterned tubular-like structures. Mechanistic studies confirmed that miR-765 decreased the levels of VEGFA, AKT1 and SRC-α transducers and exerted a negative regulation of VEGFA by specific binding to its 3‘UTR. Finally, overall survival analysis of a cohort of ovarian cancer patients (*n* = 1435) indicates that high levels of VEGFA, AKT1 and SRC-α and low miR-765 expression were associated with worst patients outcome. In conclusion, here we reported a novel hypoxamiRs signature which constitutes a molecular guide for further clinical and functional studies on the early stages of VM. Our data also suggested that miR-765 coordinates the formation of 3D channels-like structures through modulation of VEGFA/AKT1/SRC-α axis in SKOV3 ovarian cancer cells.

## Introduction

Tumor vasculogenic mimicry (VM) is a novel cancer hallmark formerly described in malignant melanoma cells which involves the formation of patterned three dimensional (3D) channels networks by tumor cells (1). These tubular networks resemble embryonic vasculogenesis, and they describe the ability of certain types of aggressive cancer cells to express endothelium-associated genes (2). Tumor VM occur *de novo* without or in combination with blood vessels formation changing our conventional acceptance that classical angiogenesis is the only means by which cancer cells acquire a nutrients supply to nourish tumors. Studies supporting these assumptions have demonstrated that *in vivo* the 3D channels contain plasm, erythrocytes and blood flow with a hemodynamics similar to those occurring in endothelial vessels (3). Evidences for VM have been found in other solid tumors and cancer cell lines such as in glioblastoma (4), breast (5, 6), prostate (7), lung (8), hepatocellular (9) and ovarian cancers (10, 11), among others. This morphologic plasticity have been associated to aggressive tumor phenotypes, increased metastasis and tumor progression of certain types of cancers. Moreover, meta-analysis studies have established a definitive association between VM with poor clinical poor prognosis in human cancer patients (12). Remarkably, tumor VM may contribute to the resistance of diverse type of tumors against anti-angiogenic therapy (13, 14). Therefore, the exploration of the multiple roles of VM in cancer hallmarks, especially in drug resistance, would broaden our knowledge and eventually ameliorate the treatment efficacy in cancer.

Cellular features underlying VM are diverse although they may summarized as follows: (i) vascular-like tubules are lined by tumor cells in combination or not with endothelial cells forming complex 3D mosaic patterns; (ii) VM cells achieve remodeling of extracellular matrix and tumor microenvironment; (iii) 3D channels assembled during VM connects with the tumor microcirculation system providing blood and supplies for tumor growth, (iv) VM provides also a perfusion route for metabolic waste; and (v) in tumor tissues VM cells showed Periodic-acid Schiff (PAS) positive and CD31 negative staining which provides a new tool for potential use in clinical practice (15). Nonetheless, *in vitro* reports on VM are still debatable because only few studies provide solid evidence of 3D tube formation (1, 16–19) or use malignant melanoma or ovarian cancer cell lines previously confirmed to form tubular 3D structures (19–21). In an outstanding paper from Owen's lab this controversy was addressed by characterizing VM *in vitro* using SKOV3, HEY and other ovarian cancer cell lines, as well as spheres and primary cultures derived from ovarian cancer ascites (19). Using dye microinjection, X-ray microtomography 3D-reconstruction, and confocal microscopy studies they confirmed that glycoprotein-rich lined 3D tubular structures are present in *in vitro* cultures and were able of conducting fluids. This study highlights the importance of confirmatory *in vitro* assays for VM, and surprisingly suggested that many of 3D cellular networks reported in the literature may not represent genuine VM (19).

Diverse molecular mechanisms and signaling pathways have been described to be involved in VM formation (22–24). Moreover, it has been described that aggressive tumor cells undergoing VM showed specific gene-expression profiles that resembles that of an undifferentiated, embryonic-like cells (2). Molecular mechanisms operating in VM have been extensively studied recently with some master regulators identified (25). For instance, hypoxia inducible factor 1-α (HIF-1α) greatly promotes VM formation in response to hypoxia as it occurs in angiogenesis (26). The role of other proteins and signaling pathways that promote cell proliferation, migration, invasion and matrix remodeling during tumor VM also has been described. These include factors such as the vascular endothelial-cadherin (VE-cadherin) (21, 27), epithelial cell kinase (EphA2) (18), phosphoinositide 3-kinase alpha (PI3K-α) (6), matrix metalloproteinase (MMPs), laminin 5 (Ln-5) γ2 chain, focal adhesion kinase (FAK) (23–25) and proto-oncogene tyrosine-protein kinase SRC-α (6). Although important advances in deciphering the molecular mechanism underlying VM, the fine-tuning modulation and the role of non-coding RNAs in the early stages of VM remains poorly understood.

During the last decades, the study of non-coding RNAs in cancer biology has exploded revealing unsuspected functions in tumorigenesis. MicroRNAs (miRNAs) are non-coding single-stranded small RNAs of 21-25 nucleotides in length that function as negative regulators of gene expression (28). MiRNAs function as guide molecules in post-transcriptional gene silencing by partially complementing with the 3′-end of target transcripts resulting in mRNA degradation or translational repression in cytoplasmic P-bodies (29). These small non-coding RNAs may target a plethora of regulatory molecules driving tumorigenesis. Recent studies showed that some miRNAs have a pivotal role in VM in diverse types of solid tumors. For instance, miR-26b targets EphA2 a VM regulator in glioma (30). In breast cancer, miR-204 exerts a fine-tuning regulation of the synergistic transduction of PI3K/AKT1/FAK mediators critical in VM formation (6). In ovarian cancer only two studies about the role of miRNAs, specifically miR-200a and miR-27b, have been reported (31, 32), indicating that detailed miRNAs functions in VM regulation in ovarian cancer remains to be elucidated. In the present investigation, we reported a novel miRNAs signature activated during the hypoxia-induced 3D channels-like networks formation in ovarian cancer cells. Also, we provide functional data suggesting a role for miR-765 in VM through regulation of VEGFA/AKT1/SRC-α axis.

## Materials and Methods

### Cell Lines

Human ovarian cancer cell line SKOV-3 was obtained from the American Type Culture Collection (ATTC HTB-77), and routinely grown in Dulbecco's modification of Eagle's minimal medium (DMEM) supplemented with 10% fetal bovine serum and penicillin-streptomycin (50 unit/ml; Invitrogen, Carlsbad, CA, USA).

### Periodic Acid Staining

3D-cultures were fixed in 4% formaldehyde in phosphate buffered solution (PBS) 1X for 30 min at room temperature. Coverslips were incubated with 0.5% periodic acid for 5 min, washed with PBS 1X for 5 min and Schiff reagent for additional 15 min. Then, cells were washed with PBS 1X for 5 min. Later they were incubated with hematoxylin for 1 min and washed in tap water for 5 min. Samples were dehydrated and mounted in coverslip using a synthetic mounting medium for microscopy.

### Three Dimensional (3D) Cultures

Experiments were performed with 70–80% confluent cell cultures. 3D cultures were prepared for confocal microscopy analysis as follow: 18 × 18 mm glass coverslips were acetone-washed, air-dried and placed in 6-well culture plates, coated with 50 μL of Matrigel per coverslip and air-dried for 60 min at room temperature. Cell cultures were trypsinized, and 60,000 cells were resuspended in 200 μL of culture medium, which was seeded on matrigel-coated coverslips. Cells were incubated at 37°C for 3 h to allow its adhesion to the matrix and then covered with 3 ml of culture medium.

### Immunofluorescence Analysis

Briefly, 3D-cultures were fixed in 4% formaldehyde in PBS 1X for 30 min at room temperature. Coverslips were incubated with 0.1% Triton X-100 for 3 min. Following washing with PBS 1X, cells were blocked for 40 min at room temperature with 0.2% BSA in PBS 1X, and incubated with Phalloidin 1X (Abcam, ab235138) for 30 min at room temperature. Stained cells were then washed with PBS 1X for 15 min and mounted for confocal microscopy.

### RNA Isolation

Total RNA was extracted using 500 μl Trizol (Invitrogen, Carlsbad, CA) for 1 × 10^4^ cells/well as described the manufacturer. RNA integrity was assessed using capillary electrophoresis system Agilent 2100 Bioanalyzer. Samples with a RNA integrity >5 were processed.

### MicroRNAs Expression Profiling

The Megaplex TaqMan Low-Density Array (TLDA) v 3.0 (Applied Biosystems, Foster City, CA) platform was used to measure the expression of 754 human specific miRNAs in parallel. Briefly, total RNA (600 ng) was retro-transcribed using stem-loop primers specific for each miRNA in order to obtain complementary DNA (cDNA) templates. Subsequently, a pre-amplification step of 12 cycles was included to increase the concentration of low-level miRNAs. The pre-amplified products were loaded into the TLDA and reactions were started using the 7900 FAST real-time thermal cycler (ABI). RNU44 and RNU48 expression was used as internal control. For statistical analysis miRNAs levels were measured by quantitative reverse transcription polymerase chain reaction (qRT-PCR) in TLDA using the comparative Ct (2ΔΔCt) method. All analyses were done using R (HTqPCR and gplots-bioconductor). The Ct raw data were determined using an automatic baseline and a threshold of 0.2. A fold change (FC) (log2 RQ) value >1.5 was used to define the differentially expressed miRNAs. An adjusted *t*-test was used to evaluate the significant differences in Ct values between groups. To identify subgroups defined by miRNA expression profiles, an unsupervised clustering analysis using Spearman correlation and average linkage was used.

### Bioinformatics Analysis

MiRNA targets were identified using TargetScan 7.0 (http://www.targetscan.org/vert_71/), and PicTar (http://www.pictar.org/) softwares. Only target genes that were predicted by the two algorithms were selected for further analysis. Gene ontology and enrichment cellular pathway analyses were performed using David tool.

### Transfection of miR-765 Mimic

MiRNA-765 mimic (AM17100 ThermoFisher), and pre-miR-negative control scramble (AM17110 ThermoFisher) were transfected in SKOV3 cells using siPORT amine transfection agent. Briefly, miR-765 (80 nM) and scramble (80 nM) were individually added to wells containing 1 × 10^4^ cells cultured in DMEM for 48 h. Then, overexpression of miR-765 was confirmed by quantitative RT-PCR at 48 h postransfection using total RNA. MiR-765-expressing cells were used for downstream analysis.

### 3D Channels-Like Networks Inhibition Assays

3D channels-like networks experiments were performed through 3D-dimensional cultures on matrigel. Firstly, SKOV3 cells (1 × 10^4^ cells/well) were transfected with pre-miR-765 (80 nM) or scramble (30 nM) negative control as previously described. The cells were cultured in 96-well plate covered with geltrex matrix (50 μl). Afterward, cells were incubated at 37°C in 5% CO_2_ atmosphere in hypoxia conditions (1% O_2_) for 48 h. Then, the formation of 3D channels formation was induced by seeding cells on matrigel and then capillary-like structures were observed under an inverted microscope (Iroscope SI-PH) and imaged during 0, 6, and 12 h. Two observers individually counted the number of branch points and tubular structures. Data were expressed as mean ±S.D. *p* < 0.05 was considered as statistically significant.

### Western Blot Assays

30 μg of whole protein extracts were separated on 12% SDS-PAGE and transferred to 0.2 μm nitrocellulose membrane (Bio-Rad) and then incubated with the following primary antibodies: anti-AKT1 (1:1000, C74H10 Cell signaling), anti-SRC-α (1:1000; sc-130124 Santa Cruz), anti-VEGFA (1:500, ab183100 abcam) and anti-GAPDH (1:1000, sc-365062 Santa Cruz). Densitometry analysis of immunodetected bands in Western blots assays were performed using the public domain myImage Analysis software.

### Luciferase Gene Reporter Assays

DNA fragments of the 3'UTR of VEGFA gene containing the predicted miR-765 binding sites were cloned into p-miR-report vector (Ambion) downstream of luciferase gene. All constructs were verified through automatic sequencing. Then, recombinant pmiR-LUC-VEGFA plasmid was transfected into SKOV3 cells using lipofectamine 2000 (Invitrogen). At 24 h after transfection, pre-miR-765 (80 nM) and scramble were co-transfected with lipofectamine RNAi max (Invitrogen). Then, 24 h after transfection firefly and *Renilla reniformis* luciferase activities were both measured by the Dual-Glo luciferase Assay (Promega) using a Fluoroskan Ascent™ Microplate Fluorometer. Firefly luciferase activity was normalized with *Renilla reniformis* luciferase.

### Kaplan Meier Analysis

Overall survival analysis using Kaplan Meier plotter for miR-765, VEGFA, AKT1, and SRC-α genes in ovarian cancer patients were evaluated as previously described (33, 34). Briefly, we used the Start KM plotter for ovarian cancer tool that use genome-wide for mRNA expression data and overall survival clinical information of cancer patients, which were downloaded from Gene Expression Omnibus GEO (Affymetrix HG-U133A, HG-U133A 2.0, and HG-U133 Plus 2.0 microarrays) and The Cancer Genome Atlas TCGA, whereas for miRNAs expression we used Start miRpower for pan-cancer as implemented in the KM plotter at the URL (http://kmplot.com/analysis/index.php?p=backgroundr). To define the prognostic value of genes the samples were split into two groups according to various quantile expression of miR-765 (*n* = 485) and VEGFA, AKT1 and SRC-α genes in ovarian cancer patients (*n* = 1435). A Kaplan-Meier survival plot compared the two patient cohorts, and the hazard ratio with 95% confidence intervals and logrank *P*-value were calculated.

### Statistical Analysis

Experiments were performed three times by triplicate and results were represented as mean ±S.D. One-way analysis of variance (ANOVA) followed by Tukey's test were used to compare the differences between means. A *p* < 0.05 was considered as statistically significant.

## Results

### MicroRNAs Modulated During Hypoxia-Induced 3D Channels-Like Structures Formation in Ovarian Cancer Cells

**Figure 1:**
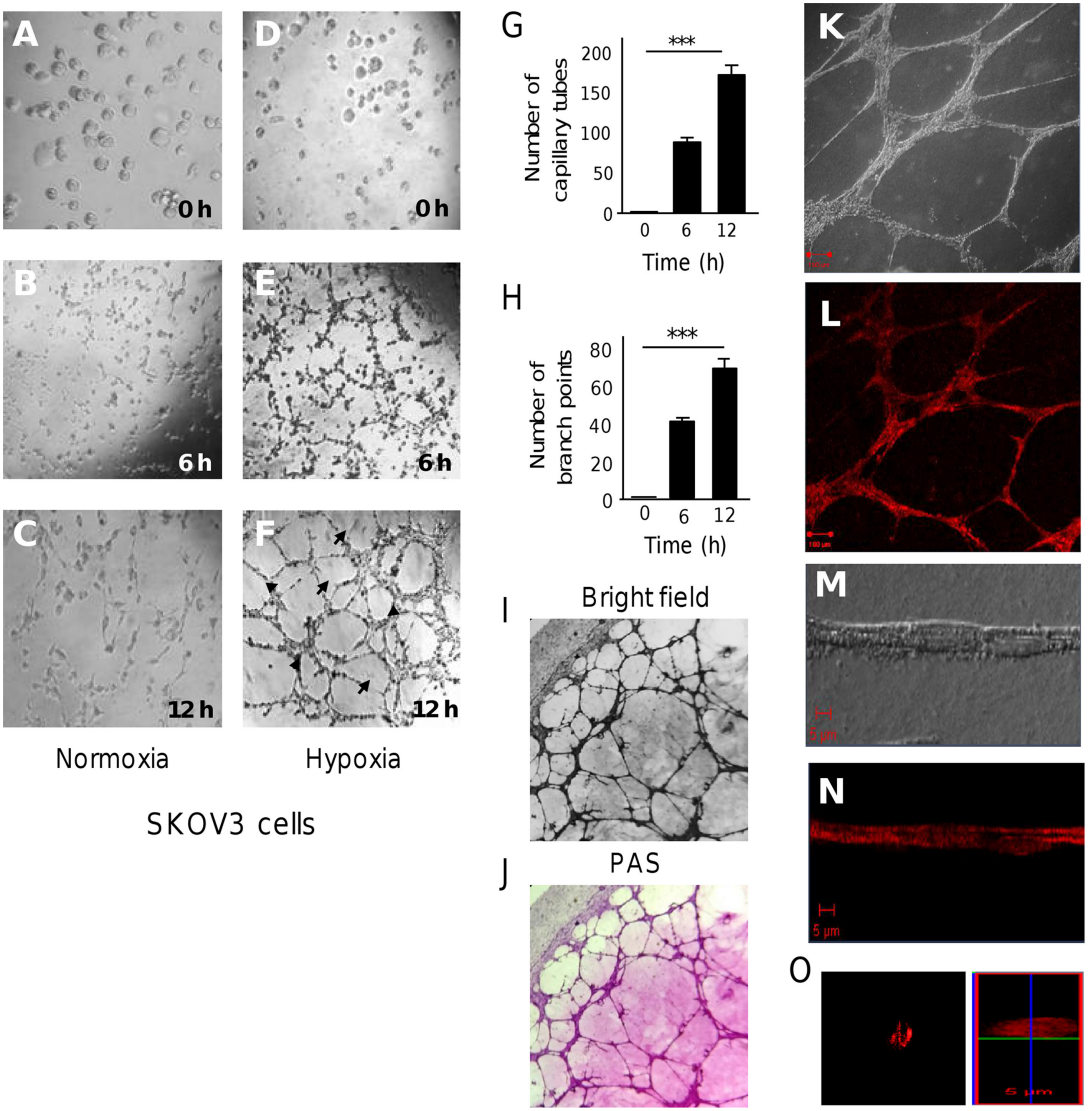
3D channels-like formation in SKOV3 ovarian cancer cells. **(A-F)** SKOV3 cells were previously incubated onto matrigel with serum free medium for 12 h (time 0), and then imaged during course of time (0–12 h) as showed in **(A-C)** normoxia and **(D-F)** hypoxia conditions. Arrows denote the capillary-like tubes. Arrowheads denote the branch points. **(G)** Graphical representation of quantification of cellular networks and **(H)** branch points number after 0, 6, and 12 h. Experiments were performed three times by triplicate and data were expressed as mean ± S.D. ^***^p < 0.001. **(I)** Bright field images (10×) and **(J)** Periodic acid-Schiff (PAS) stained images (10×) of cultures on matrigel. **(K-O)** Images of 3D-culture observed under confocal laser-scanning microscopy. Cells in **(K,M)** clear field and stained with **(L,N,O)** rhodamine-phalloidin. **(O)** Confocal microscopy Z-stack reconstruction of cellular networks.

To investigate the role of hypoxia in expression of miRNAs associated with the initial phases of vasculogenic mimicry (VM), firstly we established an *in vitro* model for three-dimensional (3D) channels-like structures formation representative of the early stages of VM. We have chosen the SKOV3 ovarian cancer cells which were previously unequivocally demonstrated to form vasculogenic mimicry *in vitro* after 4 days incubation in hypoxia (19). Here, SKOV3 cells were grown in confluent monolayers under hypoxia (1% O_2_) or normoxia conditions during 48 h. Then, cells were seeding on matrigel and incubated for 0, 6, and 12 h to track the formation of 3D capillary-like structures, which represent the stages previous to VM formation. Results showed that SKOV3 cells grown in normoxia hardly exhibited the formation of cellular networks after 6 and 12 h incubation on matrigel (Figures 1A–C). When cells were grown in hypoxia, a dramatical increase in extend of cellular networks was observed during the course of time. SKOV3 cells exhibited the typical morphologic changes indicative of 3D channels-like networks formation after 0 and 6 h incubation on matrigel (Figures 1D,E). Remarkably, after 12 h incubation a significant and gradual increase in networks was found (Figure 1F). Quantification of the number of cellular networks showed that these structures were significantly augmented from 98 ± 4 to 172 ± 7 after 6 and 12 h incubation, respectively (Figure 1G). Likewise, the number of branch points was significantly increased from 43 ± 2 to 71 ± 4 after 6 and 12 h, respectively (Figure 1H). At 12 h, positive PAS staining was found mainly along the length of the cellular networks suggesting the existence of extracellular matrix compounds (Figures 1I,J). To evaluate the potential presence of tubular structures with a hollow tube, SKOV3 cells were stained with rhodamine-phalloidin and analyzed by confocal microscopy (Figures 1K,L). Immunofluorescence images of the cellular networks showed very discrete elevated structures with tubular-like appearances as observed in bright field and red channel (Figures 1M,N). A confocal microscopy Z-stack reconstruction of 12 h old 3D-cultures of SKOV3 cells hardly showed the presence of proper tubular structures with hollow centers (Figures 1N,O). These findings indicate that after 48 h hypoxia and 12 h incubation on matrigel, no clear tubules with hollow centers were generated by SKOV3 cells. Instead of we found cellular networks which were organized and lined in a time-dependent manner.

**Figure 2:**
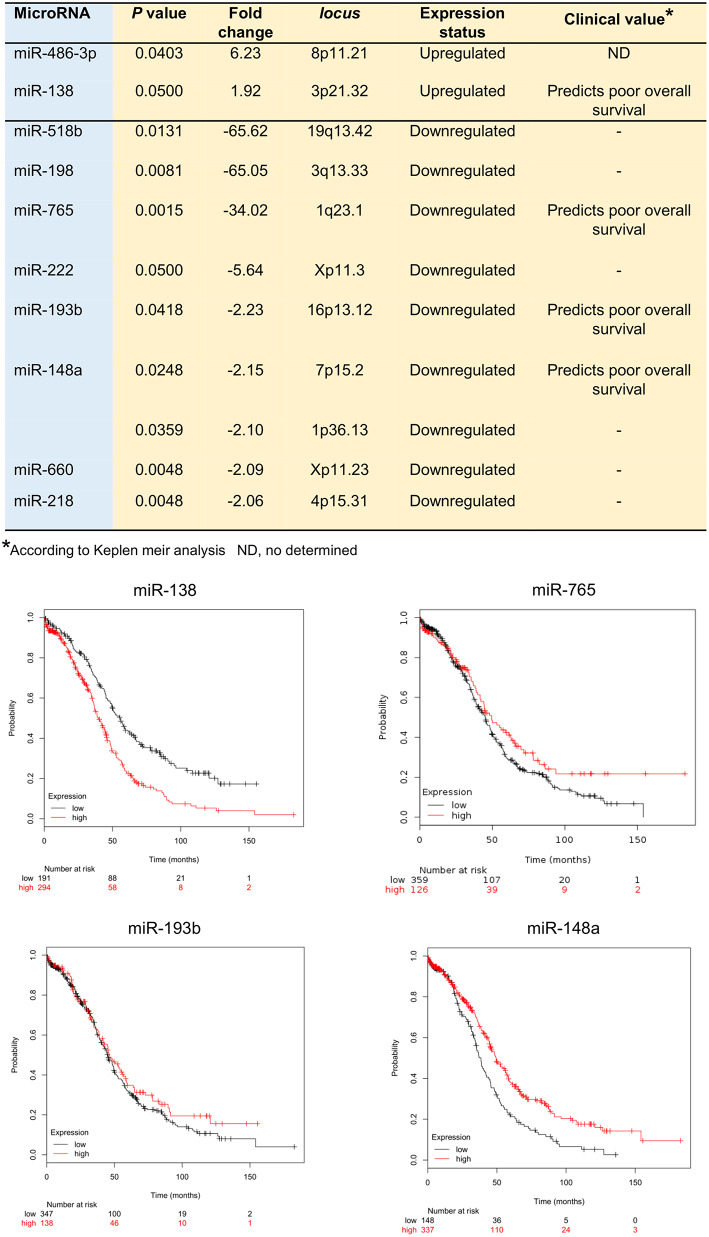
MicroRNAs deregulated in SKOV3 cells after 48 h hypoxia. **Upper Table** illustrate the hypoxamiRs regulated in SKOV3 cells. The miRNAs expression status and clinical value predicted after Kaplan Meir analysis is depicted. **Bottom Images** showed the Kaplan Meir plots for four hypoxamiRs with potential clinical value using Start miRpower for pan-cancer as implemented in the KM plotter online tool (http://kmplot.com/analysis/index.php?p=backgroundr).

In order to identify the set of miRNAs regulated by hypoxia (hypoxamiRs) before VM formation, we profiled 667 mature miRNAs using Taq Man Low Density Arrays (TLDAs) after 48 h hypoxia. Our results showed that 11 unique hypoxamiRs were significantly modulated (FC>1.5; *p* < 0.05) in SKOV3 cells. Of these 9 miRNAs were downregulated (miR-765, miR-660, miR-218, miR-198, miR-518b, miR-148a, miR-1290, miR-193b, miR-222) and 2 upregulated (miR-486-3p, miR-138) in comparison to control cells grown without hypoxia (time 0) (Figure 2). Next, we were wondering if expression levels of the set of modulated miRNAs may have clinical implications in ovarian cancer. Therefore, we performed overall survival analysis using Kaplan Meier tool (Start miRpower pan-cancer) which utilize genome-wide transcriptome data and overall survival clinical information from a large cohort of ovarian cancer patients (*n* = 485) with a follow-up of 180 months as described in material and methods (33, 34). To define the prognostic value of genes the samples were split into two groups according to quantile expression of miRNAs. A Kaplan-Meier survival plot compared the two patient cohorts, and the hazard ratio with 95% confidence intervals and logrank *P*-value were calculated. Results showed that high expression of miR-138 (HR = 1.80, logrank *P* = 5.3e-07) and low levels of miR-765 (HR = 0.77, logrank *P* = 0.05), miR-193b (HR = 0.86, logrank *P* = 0.25), and miR-148a (HR = 0.63, logrank *P* = 0.0001) genes were associated to low overall survival of ovarian cancer patients (Figure 2).

### HypoxamiRs Regulate Cellular Pathways Associated With Cancer

**Figure 3:**
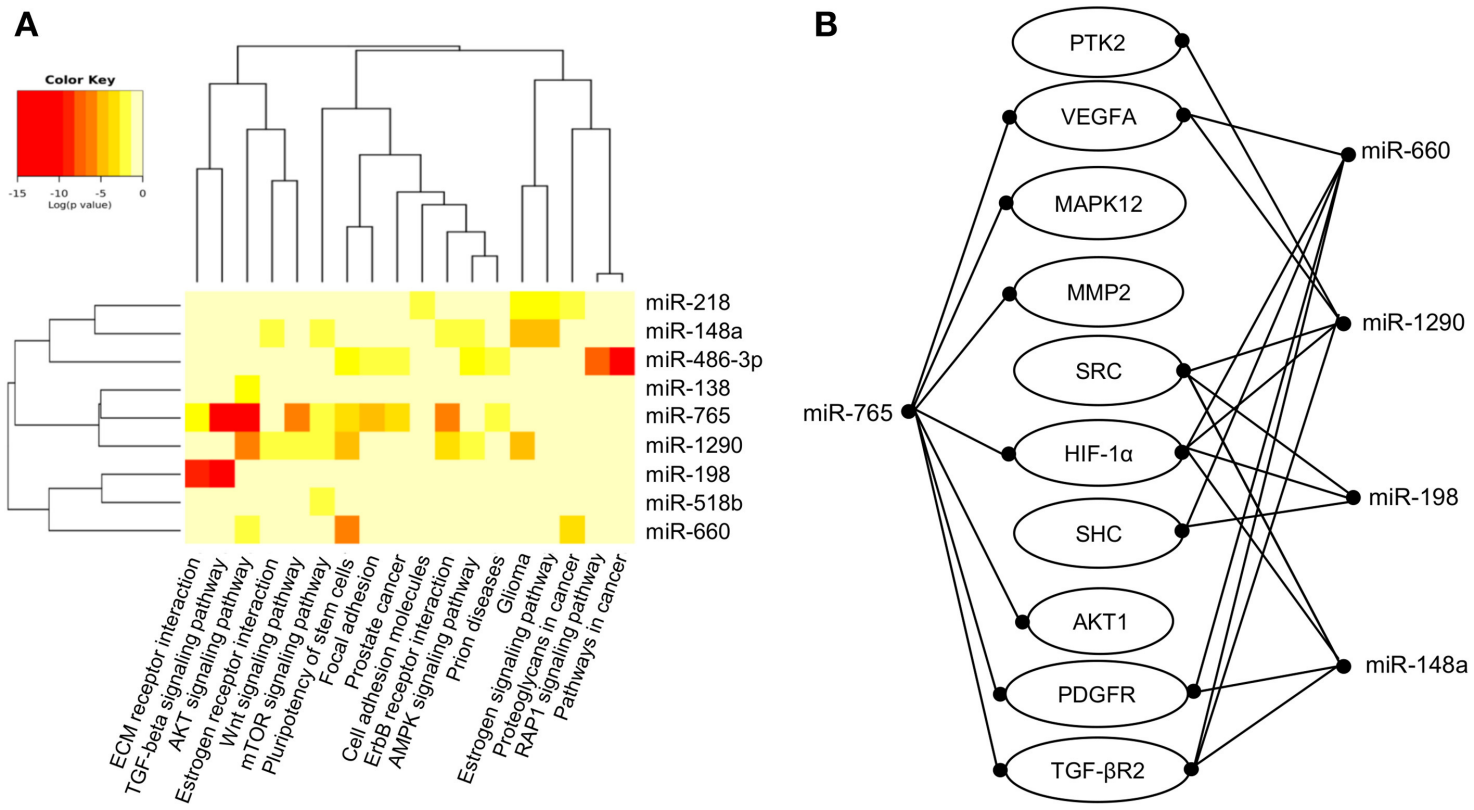
Core miRNA/mRNA interaction networks. **(A)**. Supervised hierarchical clustering of signaling pathways affected by deregulated miRNAs. MicroT-CDS function and Euclidean correlation were used; a *p* < 0.05 was considered to identify significantly differentially expressed miRNAs in SKOV3 cells after 48 h in hypoxia. Columns display the clustering of cellular pathways. Rows indicate the clustering of miRNAs names, and pathways are denoted at bottom. **(B)** Illustration depicts the modulated miRNAs after 48 h of hypoxia and predicted target mRNAs involved in angiogenesis and vasculogenic mimicry.

Predictive analysis of the set of regulated hypoxamiRs suggested that they might impact common cellular processes and signaling pathways related with tumorigenesis (Figure 3A). The signaling pathways enriched were TGF-β, WNT, mTOR, AMPK, estrogen receptor and RAP1. Computational predictions also indicated that these miRNAs may target a number of genes involved in VM and angiogenesis including HIF-1A, HIF-1AN, HIF-3A, PTGFRN, AKT1, VEGFA, VEGFB, VEGFC, PDGFR, TGF-βR2, MMP2, PTK2, SRC, SHC3, and GRB2, among others (Table 1). In particular, we focused in miR-765 for further functional analysis because: (i) it was severely downregulated after hypoxia (FC < 32.02; *p* < 0.05), (ii) it was predicted to target a number of genes involved in VM (Figure 3B), and (iii) there is no reports about the functions of miR-765 in ovarian cancer neither in tumor VM.

**Table 1:** Modulated microRNAs after 48 h hypoxia in SKOV3 ovarian cancer cells and predicted targets with functions associated to cancer.

**MicroRNAs**	**Predict target**	**Protein name^a^**	**Functions**	**References**
**UPREGULATED**				
miR-486-3p	HIF1AN	Hypoxia inducible factor 1 alpha subunit inhibitor	Oxygen sensor	Kang et al. (35)
	SRCIN1	SRC kinase signaling inhibitor 1	Inhibitor of AKT/RAS pathway	
miR-138	FAM13	Fas apoptotic inhibitory molecule 3	Inhibitor of RAS pathway	Kang et al. (35)
	PTGFRN	Prostaglandin F2 receptor inhibitor	Inhibitor of angiogenesis, VM	Colin et al. (36)
	HIF1AN	Hypoxia inducible factor 1 alpha subunit inhibitor	Oxygen sensor	
**DOWNREGULATED**				
miR-765	VEGFA	Vascular endothelial growth factor A	Angiogenesis, proliferation, VM	Chen et al. (37)
	AKT1	RAC-alpha serine/threonine-protein kinase	Angiogenesis, proliferation, migration, VM	Rana et al. (38)
	HIF-3A	Hypoxia inducible factor 3 alpha	Angiogenesis, VM	Li et al. (39)
	PDGFR	Platelet-derived growth factor receptor	Proliferation, angiogenesis, migration	Wei et al. (40)
	TGFBR2	Transforming growth factor, beta receptor II	Proliferation, differentiation, angiogenesis, VM	Salinas-Vera et al. (6)
	MMP2	Matrix metallopeptidase 2	Angiogenesis, metastasis, VM	Ando et al. (41)
				Cuomo et al. (42)
				Avril et al. (43)
				Thijssen et al. (44)
				Plantamura et al. (45)
				Khalkhali-Ellis et al. (46)
				Kang et al. (47)
				Liang et al. (48)
miR-660	VEGFA	Vascular endothelial growth factor A	Angiogenesis, proliferation, VM	Luengo-Gil et al. (49)
	SRC	Proto-oncogene tyrosine-protein kinase	Proliferation, migration, VM	Salinas-Vera et al. (6)
	HIF-1A	Hypoxia inducible factor 1, alpha	Angiogenesis, VM	Jaraíz et al. (50)
	TGFBR2	Transforming growth factor, beta receptor II	Proliferation, differentiation, VM.	Chen et al. (37)
	PDGFR2	Platelet-derived growth factor receptor	Proliferation, differentiation, VM	Rana et al. (38)
				Plantamura et al. (45)
				Khalkhali-Ellis et al. (46)
				Avril et al. (43)
				Thijssen et al. (44)
miR-218	SHC1	SHC-transforming protein 1	Proliferation, angiogenesis, VM	Salinas-Vera et al. (6)
	CDH8	Cadherin-8	Migration	Thomas et al. (51)
				Memi et al. (52)
miR-198	SRC	Proto-oncogene tyrosine-protein kinase	Proliferation, migration, VM	Salinas-Vera et al. (6)
	SHC1	SHC-transforming protein 1	Proliferation, angiogenesis, VM	Jaraíz et al. (50)
	HIF-3A	Hypoxia inducible factor 3, alpha subunit	Angiogenesis, VM	Thomas et al. (51)
	PTK2	Focal adhesion kinase 1	Proliferation, migration, VM	Suen et al. (53)
				Ando et al. (41)
				Cuomo et al. (42)
miR-518b	MAPK1	Mitogen-activated protein kinase 1	Angiogenesis, proliferation, VM	Wei et al. (40)
	TGFBR2	Transforming growth factor, beta receptor II	Proliferation, differentiation, angiogenesis, VM	Flum et al. (54)
				Plantamura et al. (45)
				Khalkhali-Ellis et al. (46)
miR-148a	TGFBR2	Transforming growth factor, beta receptor II	Proliferation, differentiation, angiogenesis, VM	Plantamura et al. (45)
	MMP16	Matrix metallopeptidase 16	Angiogenesis, metastasis	Khalkhali-Ellis et al. (46)
	HIF-3A	Hypoxia inducible factor 3 alpha subunit	Angiogenesis, VM	Kang et al. (47)
				Li et al. (55)
				Ando et al. (41)
				Cuomo et al. (42)
miR-1290	VEGFA	Vascular endothelial growth factor A	Angiogenesis, proliferation, VM	Chen et al. (37)
	PTK2	Focal adhesion kinase 1	Proliferation, migration, VM	Rana et al. (38)
	SRC	Proto-oncogene tyrosine-protein kinase	Proliferation, migration, VM	Luengo-Gil et al. (49)
	HIF-1A	Hypoxia inducible factor 1 alpha	Angiogenesis, VM	Salinas-Vera et al. (6)
	TGFBR2	Transforming growth factor, beta receptor II	Proliferation, differentiation, angiogenesis, VM	Jaraíz et al. (50)
				Chen et al. (37)
				Rana et al. (38)
				Plantamura et al. (45)
				Khalkhali-Ellis et al. (46)
miR-193b	SHC3	SHC-transforming protein 3	Proliferation, angiogenesis, VM	Liu Y et al. (56)
	GRB2	Growth factor receptor-bound protein 2	Proliferation, angiogenesis, VM	Salinas-Vera et al. (6)
	CDH4	Cadherin 4, type 1	Angiogenesis, VM	Zhang et al. (57)
	HIF3A	Hypoxia inducible factor 3 alpha subunit	Angiogenesis, VM	Xie et al. (58)
				Ando et al. (41)
				Cuomo et al. (42)
miR-222	VEGFB	Vascular endothelial growth factor B	Angiogenesis, proliferation, VM	Chen et al. (37)
	VEGFC	Vascular endothelial growth factor C	Angiogenesis, proliferation, VM	Rana et al. (38)
	SHC4	SHC-transforming protein 4	Proliferation, angiogenesis, VM	Ikeda et al. (59)
	HIF1A	Hypoxia inducible factor 1, alpha subunit	Angiogenesis, VM	Thomas et al. (51)
				Suen et al. (53)
				Chen et al. (37)
				Rana et al. (38)

a *Uniprot database name; VM, vasculogenic mimicry*.

### Hypoxia-Suppressed miR-765 Inhibits Channels-Like Networks Formation

**Figure 4:**
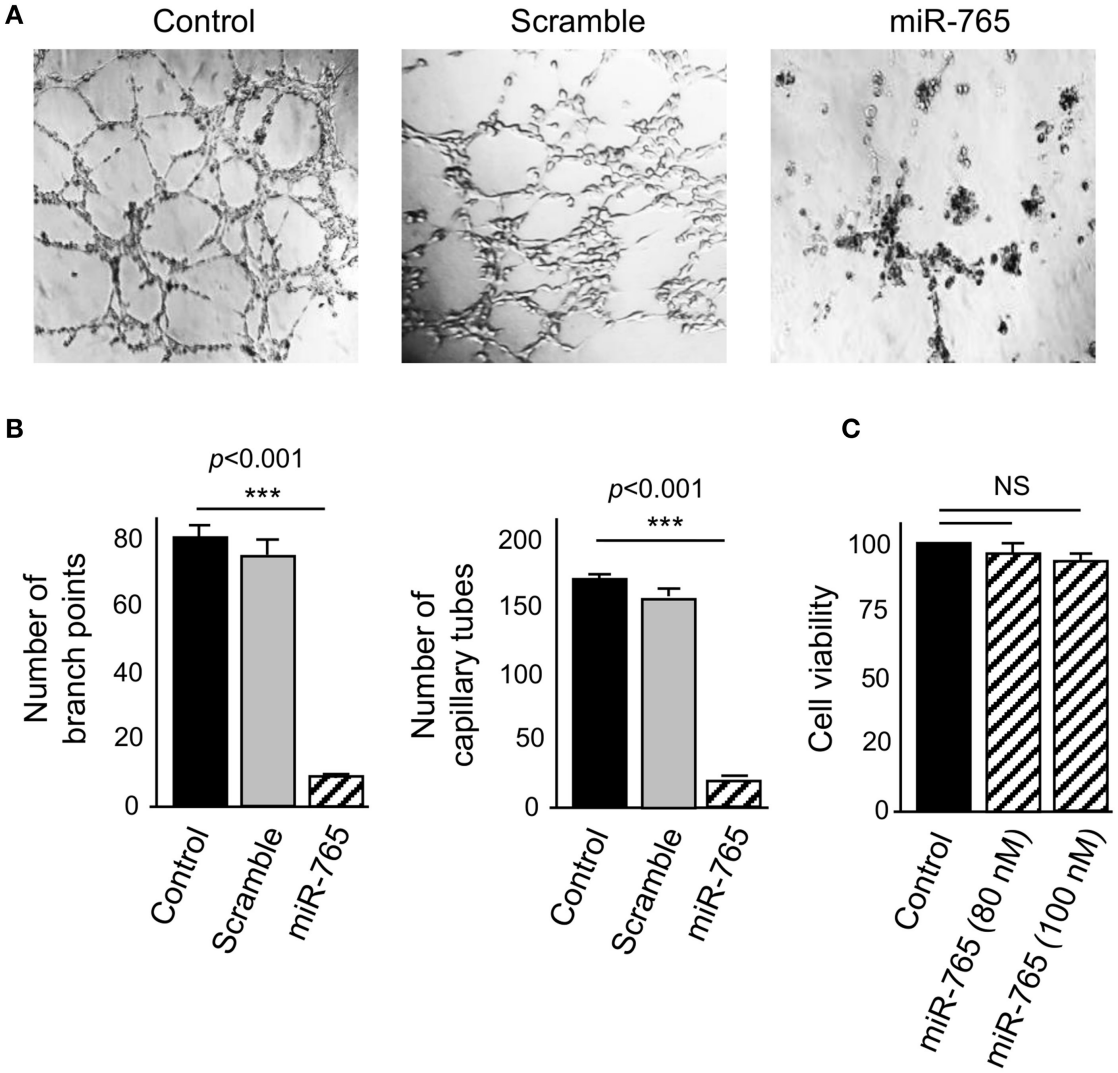
miR-765 inhibits hypoxia-induced 3D channels-like structures. **(A)** 3D channels-like structures of SKOV3 cells transfected with miR-765 mimics (right panel), scramble (middle panel) and no-transfected control cells (left panel) and grown for 48 h in hypoxia and then 12 h in matrigel. **(B)** Graphical representation of the number of branch points and capillary-like channels from **(A)**. **(C)** Cell viability assays of SKOV3 cells transfected with increasing concentrations of miR-765. Experiments were performed by three times by triplicate and data were expressed as mean ± S.D. ^***^p < 0.001. NS, non-significant.

To examine the functional role of miR-765 on 3D channels-like networks, we restored its expression in SKOV3 cells by transfection of specific RNA mimics. Then, 3D channels-like networks formation was induced by 48 h hypoxia as described before. Non-transfected and scramble-treated cells were included as controls. Interestingly, ectopic restoration of miR-765 produced a dramatic inhibition of 3D channels-like networks formation (Figure 4A). A significant reduction of the number of branch points (up to 85%) and capillary tubes (up to 92%) were found in miR-765-transfected cells in comparison to control cells (Figure 4B). To discard pleiotropic effects of miR-765 overexpression in cell survival of ovarian cancer cells, we performed cell viability assays. Data showed no significant changes in viability of miR-765-expressing SKOV3 cancer cells at the tested concentrations which indicate that the effect of miR-765 in 3D channels-like networks impairment was specific (Figure 4C).

### MiR-765 Downregulates VEGFA, AKT1 and SRC-α and Directly Target VEGFA

**Figure 5:**
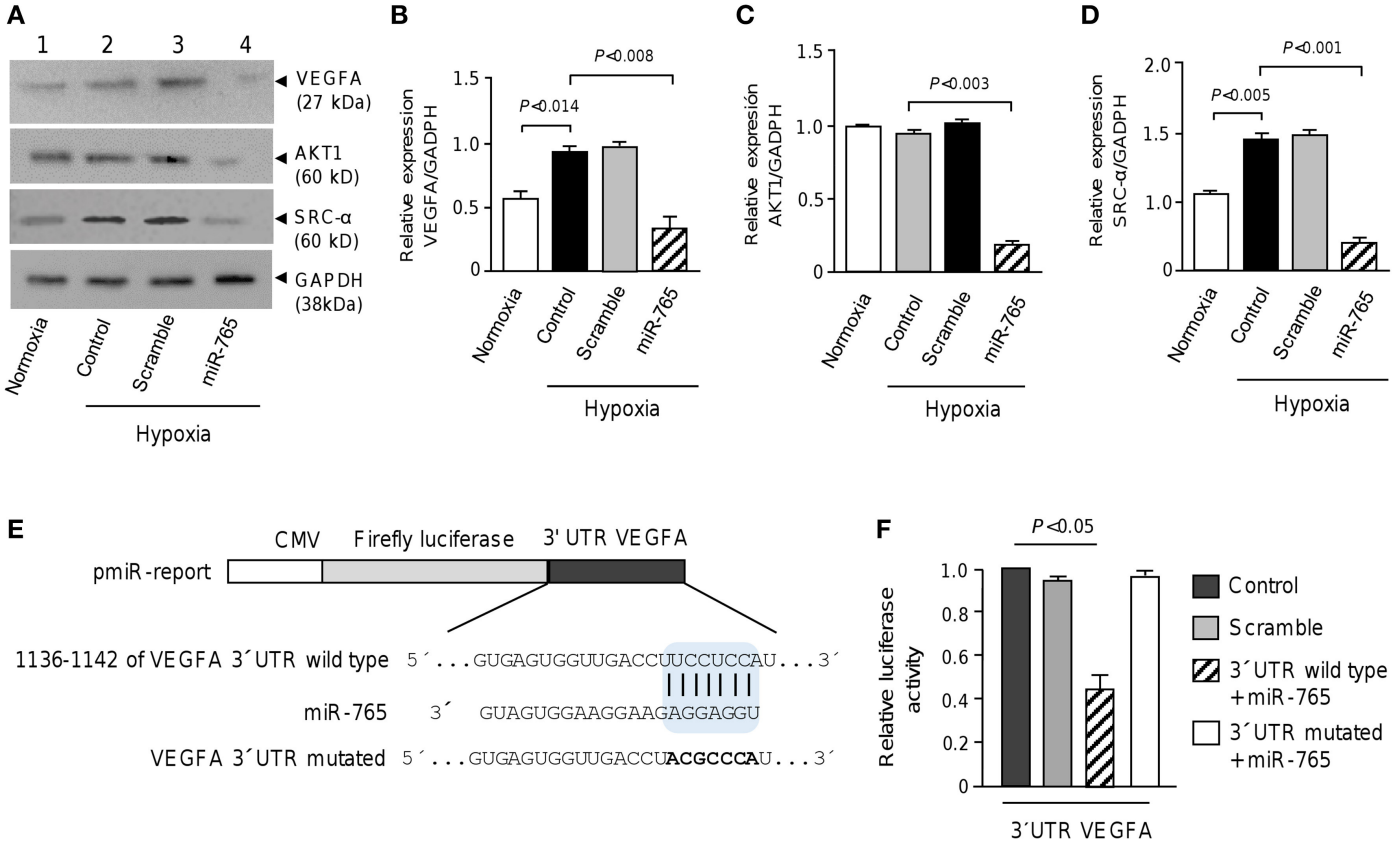
miR-765 downregulates VEGFA, AKT1 and SRC-α proteins and target VEGFA**. (A)** Immunoblots of whole proteins extracts (30 μg) from SKOV3 cells grown in normoxia or hypoxia (48 h) using specific antibodies against VEGFA, AKT1, and SRC-α. GADPH was used as loading control. Lane 1, SKOV3 cells in normoxia; lane 2, non-transfected control cells and incubated in hypoxia; lane 3, cells transfected with scramble control and incubated in hypoxia; lane 4, cells transfected with miR-765 mimics and incubated in hypoxia. **(B–D)** Densitometric quantification of immunodetected bands in panel A. Experiments were performed by triplicate and data were expressed as mean ± S.D. **(E)** Schematic representation of p-miR report construct containing the 3′UTR of VEGFA gene cloned downstream of luciferase gene. Seed sequence is indicated in colored blue box. Point mutations in the miR-765 binding sites of 3′UTR of VEGFA gene is denoted in bold. Mutations in seed sequence are denoted in bold letters. **(F)** Luciferase assays in SKOV3 cells transfected with miR-765 mimics and wild type or mutated constructs described in panel E. Cells transfected with p-miR report plasmid alone or with scramble were used as controls. Data represent the mean ± S.D. of three independent experiments.

Because the bioinformatics predictions of gene targets suggested that several signaling pathways such as VEGFA, AKT, and SRC/FAK, could be affected in SKOV3 cells transfected with miR-765, we proceed to evaluate the changes in expression of the aforementioned proteins using available antibodies in Western blot assays (Figure 5). Results showed that VEGFA protein was expressed at low levels in cells cultured under normoxia conditions, but its expression was significantly increased under hypoxia. Moreover, we observed a significant decrease in VEGFA levels in SKOV3 cells transfected with miR-765 mimics in comparison to non-treated and scramble transfected controls cells (Figures 5A,B). Likewise, a significant decrease in both SRC-α and AKT1 levels was found in cells transfected with miR-765 mimics in comparison to control cells (Figures 5A,C,D). No significant changes were observed in GADPH levels used as control. Computational predictions also showed that miR-765 may target a number of protein-encoding genes with known roles in VM. Of these, we focused in the study of VEGFA as it was downregulated by miR-765 and it contain a potential miR-765 binding site at 3′UTR (Figure 5E). To corroborate whether miR-765 can exert posttranscriptional repression of VEGFA, we performed luciferase reporter assays. A DNA fragment corresponding to 3′UTR of VEGFA was cloned downstream of the luciferase-coding region of pmiR-LUC vector (Figure 5E). In addition, a mutated version of the miR-765 binding site at the VEGFA 3'UTR was included as a plasmid control. Data showed that ectopic expression of miR-765 and co-transfection of recombinant VEGFA 3′UTR wild type plasmid into SKOV3 cells resulted in a significant reduction of the relative luciferase activity in comparison with controls (Figure 5B). In addition, when mutated sequence was assayed no significant changes in luciferase activity were found. Altogether these data confirmed that VEGFA is a novel target of miR-765.

### Expression Levels of miR-765, VEGFA, AKT1, and SRC-α Correlate With Poor Patient's Outcome

**Figure 6:**
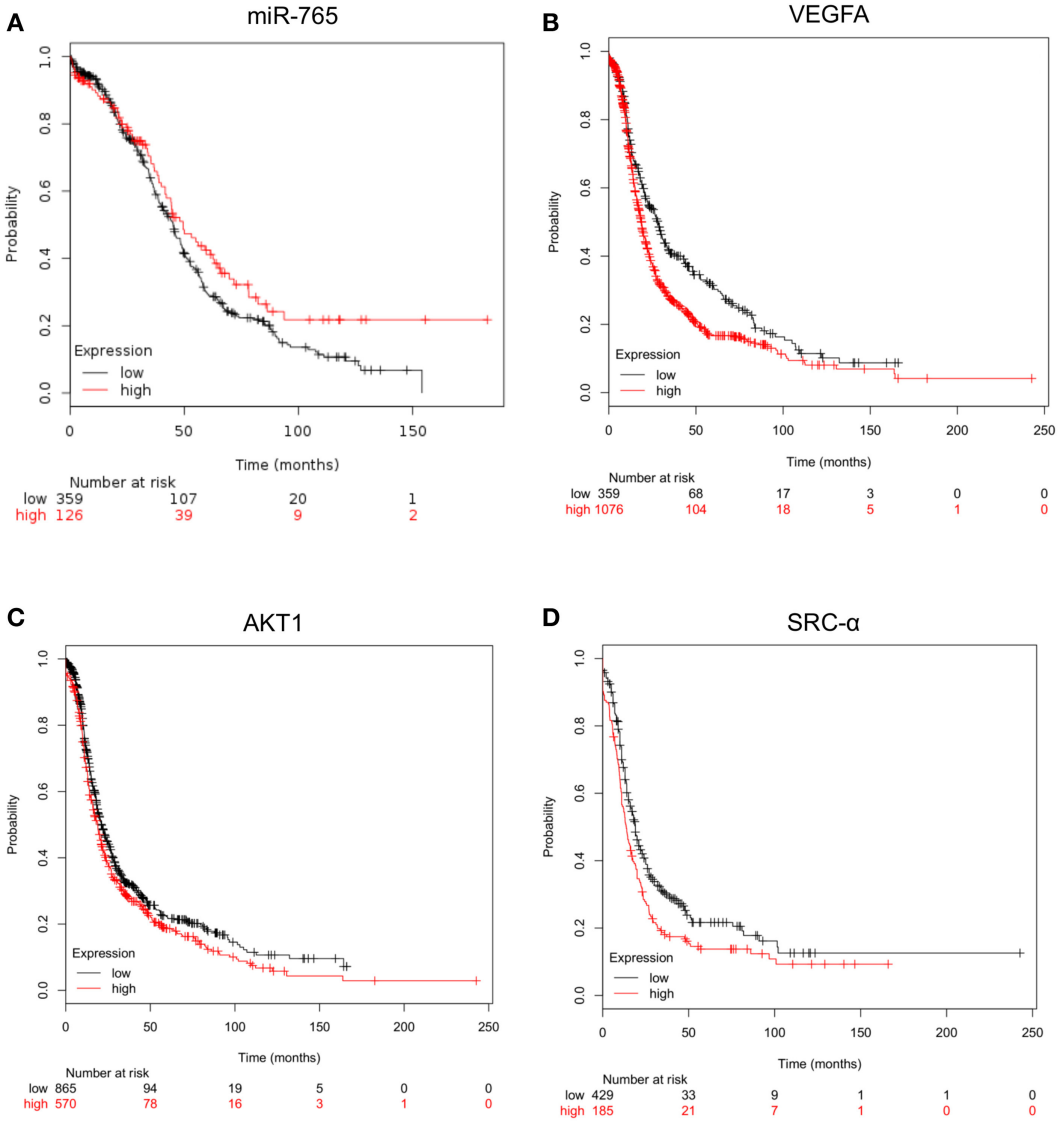
Kaplan-Meier curves for overall survival according the expression of miR-765, VEGFA, AKT1 and SRC-α. Overall survival analysis using Kaplan Meier plotter for **(A)** miR-765, **(B)** VEGFA, **(C)** AKT1, and **(D)** SRC-α genes. Start KM plotter for ovarian cancer tool used genome-wide for mRNA expression data and overall survival clinical information of cancer patients, which were downloaded from Gene Expression Omnibus GEO (Affymetrix HG-U133A, HG-U133A 2.0, and HG-U133 plus 2.0 microarrays) and The Cancer Genome Atlas TCGA, whereas for miRNAs expression we used Start miRpower for pan-cancer as implemented in the KM plotter. Samples were split into two groups according to various quantile expression of miR-765 (*n* = 485) and VEGFA, AKT1 and SRC-α genes in ovarian cancer patients (*n* = 1435). Kaplan-Meier survival plots compared the two patient cohorts, and the hazard ratio with 95% confidence intervals and logrank *P*-value were calculated.

Then we were wondering if changes in expression levels of miR-765, VEGFA, AKT1 and SRC-α have clinical implications in ovarian cancer. Thus, we performed overall survival analysis using Start Kaplan Meier plotter for ovarian cancer which use genome-wide transcriptome data and overall survival clinical information from a large cohort of ovarian cancer patients (*n* = 1485) with a mean follow-up of 170 months. To define the prognostic value of genes the samples were split into two groups according to various quantile expression of VEGFA, AKT1 and SRC-α genes. A Kaplan-Meier survival plot compared the two patient cohorts, and the hazard ratio with 95% confidence intervals and logrank *P*-value were calculated. Results showed that low levels of miR-765 (HR = 0.77, logrank *P* = 0.05) and high expression of its targets VEGFA (HR = 1.38, logrank *P* = 1.8e-05), AKT1 (HR = 1.19, logrank *P* = 0.0071), and SRC-α (HR = 1.39, logrank *P* = 0.000092) signaling genes were associated to low overall survival of ovarian cancer patients (Figure 6).

## Discussion

Tumor VM is a highly orchestrated cellular mechanism in which highly aggressive and metastatic tumor cells form vascular-like 3D networks to provide an efficient and functional fluid-conducting system for blood and oxygen supply, as an alternative to classical vasculogenesis. This morphologic plasticity is associated to high aggressiveness, increased metastasis and tumor progression of certain types of cancers. In clinical VM has been related with low overall survival and resistance to current anti-angiogenic therapies (60). Remarkably, tumor VM can be potentially targeted by novel therapeutic agents, thus currently diverse investigations in the search of novel VM regulators are undergoing. In order to contribute with the understanding of the role of small non-coding RNAs in the molecular mechanisms responsible for VM, here we have uncovered a novel set of miRNAs modulated at the early onset of hypoxia-induced 3D channels-like structures formation, previous to the proper formation of tubules indicative of VM in SKOV3 cells. We fist set-up an *in vitro* model, and using PAS staining we confirmed that SKOV3 cells efficiently form 3D channels-like networks in agreement with other studies (16, 61–63). It's important to note that the tubular-like structures we have analyzed here, may not reflect VM properly, but they represents the early stages of VM and the morphological and transcriptional programs activated by 48 h hypoxia, previous to VM appearance. It's important to remarks the urgency of confirmatory *in vitro* assays for proper VM in the different types of cancer, as a recent report (19) surprisingly suggested that many of structures reported in the literature at early times of hypoxia may not represent VM, as we can confirms in the present study.

Hypoxia is an important activator of VM, thus we decided to search for the miRNAs regulated by hypoxia (hypoxamiRs) during initial stages of VM, as it remains largely unknown in ovarian cancer. Our results showed that 11 hypoxamiRs were significantly modulated. Of these 9 miRNAs were downregulated (miR-765, miR-660, miR-218, miR-198, miR-518b, miR-148a, miR-1290, miR-193b, miR-222) and 2 upregulated (miR-486-3p, miR-138) (Figure 2). Interestingly, high expression of miR-138 and low levels of miR-765, miR-193b, and miR-148a genes were associated to low overall survival suggesting a potential clinical value in ovarian cancer patients (Figure 2). However, we cannot drawn a solid connection between outcome and VM in patients, as we have collected the clinical data from KMplot databases, and unfortunately no VM presence/absence data is available for the cohort of patients analyzed here. Thus, we have limited the conclusions only to a correlation between miRNAs regulated by hypoxia and the overall survival. On the other hand, several of the regulated miRNAs have been previously associated with tumorigenesis in diverse types of cancer. For instance, miR-660 was reported as downregulated in in lung cancer patients and its transient and stable overexpression using RNA mimics reduced migration, invasion, and proliferation properties and increased apoptosis in p53 wild-type lung cancer cells (64). Likewise, miR-218-5p expression was lower in cervical cancer tumors in comparison with normal tissues. MiR-218-5p suppressed the progression of cervical cancer via *LYN*/NF-κB signaling pathway (65). In addition, miR-138 promotes cell proliferation and invasion on colorectal cancer (66), and it contributes to resistance to therapy in multiple myeloma and non-small cell lung cancer (67, 68). Of the set of regulated hypoxamiRs, we focused in the study of functional relationships between miR-765 and 3D channels-like formation. Recently, miR-765 have been reported as upregulated or downregulated in diverse types of malignancies such as esophageal squamous cell carcinoma (69), melanoma (70), osteosarcoma (71), oral squamous cancer (72) and hepatocellular carcinoma (73). Nevertheless, miR-765 functions in ovarian cancer and tumor VM remains largely unknown. Our data showed that the ability of SKOV3 cells to develop 3D channels-like structures formation under hypoxia was significantly reduced after transfection of miR-765 mimics. This may be explained as the target predictions indicate that miR-765 may regulate genes associated to the cell proliferation, matrix remodeling, migration, and invasion, angiogenesis, and VM formation. Indeed, we demonstrated that miR-765 was able to downregulate the VEGFA, AKT1 and SRC-α signaling transducer critical in VM. Also important is the fact that expression of miR-765 and its aforementioned gene targets have a potential clinical value as its deregulation was associated with worst outcome in ovarian cancer patients (Figure 6). Main limitations of the present study are denoted by the use of a single cell model, which however, permit us to delineate important conclusions about the hypoxamiRs modulated in SKOV3 cells, and guide us to the analysis of miR-765 and its role in 3D channels-like structures formation. Nonetheless, we understand the need to extend our initial findings in additional ovarian cancer cell lines in future studies. Also, a limitation of the present study is that we specifically analyzed here the early stages of VM (after 48 h hypoxia); thus the potential role of the revealed miRNAs signature at later stages of proper VM is unknown. Taken altogether, we propose that miR-765 may regulate 3D channels-like structures formation through both direct and indirect targeting of signaling transducers. Also, we suggested that miR-765 could impair VEGFA by direct binding to VEGFA and AKT1; as well as by indirect downregulation of SRC-α which in turn may block the VEGFA/AKT1 signaling transduction. In conclusion, in the current work we provide a novel set of regulated hypoxamiRs and experimental data supporting an unexpected role for VEGFA/AKT1/SRC-α axis in 3D channels-like structures formation in SKOV3 cells. As novel therapies targeting hypoxic cancer cells are needed to improve therapy treatment of cancer, we consider that our data are relevant and deserves further *in vivo* validation.

## Author Contributions

YS-V, RG-V, OH, EC-S, and MR-H conducted all the experiments. JG-B performed the microRNAs profiling. CV-C performed the confocal microscopy. JC-C provide advice in cell cultures. DG-R, ER-G, HA, and AC-P contributed to experimental design, intellectual input, and interpreting data. CL-C and LM wrote the manuscript.

### Conflict of Interest Statement

The authors declare that the research was conducted in the absence of any commercial or financial relationships that could be construed as a potential conflict of interest.
