# Corrigendum: HypoxamiRs Profiling Identify miR-765 as a Regulator of the Early Stages of Vasculogenic Mimicry in SKOV3 Ovarian Cancer Cells

**DOI:** 10.3389/fonc.2020.00889

**Published:** 2020-06-02

**Authors:** Yarely M. Salinas-Vera, Dolores Gallardo-Rincón, Raúl García-Vázquez, Olga N. Hernández-de la Cruz, Laurence A. Marchat, Juan Antonio González-Barrios, Erika Ruíz-García, Carlos Vázquez-Calzada, Estefanía Contreras-Sanzón, Martha Resendiz-Hernández, Horacio Astudillo-de la Vega, José L. Cruz-Colin, Alma D. Campos-Parra, César López-Camarillo

**Affiliations:** ^1^Posgrado en Ciencias Genómicas, Universidad Autónoma de la Ciudad de Mexico, Mexico City, Mexico; ^2^Laboratorio de Medicina Translacional y Departamento de Tumores Gastro-Intestinales, Instituto Nacional de Cancerología, Mexico City, Mexico; ^3^Programa en Biomedicina Molecular y Red de Biotecnología, Instituto Politécnico Nacional, Mexico City, Mexico; ^4^Laboratorio de Medicina Genómica, Hospital Regional 1 de Octubre ISSSTE, Mexico City, Mexico; ^5^Departamento de Infectómica y Patogénesis Molecular, CINVESTAV-IPN, Mexico City, Mexico; ^6^Laboratorio de Investigación Translacional en Cáncer y Terapia Celular, Hospital de Oncología, Centro Médico Nacional Siglo XXI, Mexico City, Mexico; ^7^Subdirección de Investigación Básica, Instituto Nacional de Medicina Genómica, Mexico City, Mexico; ^8^Laboratorio de Genómica, Instituto Nacional de Cancerología, Mexico City, Mexico

**Keywords:** ovarian cancer, vasculogenic mimicry, hypoxia, miR-765, VEGFA

In the original article there was a mistake in the title. This article deals with “miR-765” and not “miR-745.” The correct title is above.

The authors apologize for this error and state that this does not change the scientific conclusions of the article in any way. The original article has been updated.

